# General versus sports-specific injury prevention programs in athletes: A systematic review on the effects on performance

**DOI:** 10.1371/journal.pone.0221346

**Published:** 2019-08-29

**Authors:** Ashley Plummer, Hendrik Mugele, Kathrin Steffen, Josefine Stoll, Frank Mayer, Juliane Müller

**Affiliations:** 1 Clinical Exercise Science, University of Potsdam, Department of Sport and Health Sciences, Potsdam, Germany; 2 Department of Sport Science, University of Innsbruck, Innsbruck, Austria; 3 Oslo Sports Trauma Research Center, Oslo, Norway; 4 University Outpatient Clinic, Professorship of Sports Medicine and Orthopedics, University of Potsdam, Potsdam, Germany; 5 Professorship for Physiotherapy: Exercise Science and Applied Biomechanics, Trier University of Applied Science, Trier, Germany; University of Brasilia, BRAZIL

## Abstract

**Introduction:**

Injury prevention programs (IPPs) are an inherent part of training in recreational and professional sports. Providing performance-enhancing benefits in addition to injury prevention may help adjust coaches and athletes’ attitudes towards implementation of injury prevention into daily routine. Conventional thinking by players and coaches alike seems to suggest that IPPs need to be specific to one’s sport to allow for performance enhancement. The systematic literature review aims to firstly determine the IPPs nature of exercises and whether they are specific to the sport or based on general conditioning. Secondly, can they demonstrate whether general, sports-specific or even mixed IPPs improve key performance indicators with the aim to better facilitate long-term implementation of these programs?

**Methods:**

PubMed and Web of Science were electronically searched throughout March 2018. The inclusion criteria were randomized control trials, publication dates between Jan 2006 and Feb 2018, athletes (11–45 years), injury prevention programs and included predefined performance measures that could be categorized into balance, power, strength, speed/agility and endurance. The methodological quality of included articles was assessed with the Cochrane Collaboration assessment tools.

**Results:**

Of 6619 initial findings, 22 studies met the inclusion criteria. In addition, reference lists unearthed a further 6 studies, making a total of 28. Nine studies used sports specific IPPs, eleven general and eight mixed prevention strategies. Overall, general programs ranged from 29–57% in their effectiveness across performance outcomes. Mixed IPPs improved in 80% balance outcomes but only 20–44% in others. Sports-specific programs led to larger scale improvements in balance (66%), power (83%), strength (75%), and speed/agility (62%).

**Conclusion:**

Sports-specific IPPs have the strongest influence on most performance indices based on the significant improvement versus control groups. Other factors such as intensity, technical execution and compliance should be accounted for in future investigations in addition to exercise modality.

## Introduction

In the past decade, youth participation in organized sport has increased 21% resulting in approximately 30 million youth amateur athletes in the US alone [1]. This observation is likely driven by numerous health initiatives encouraging the many physical and psychosociological benefits of physical activity [2]. Aside from the clear benefits of participation, >30% of injuries among adolescents are sport-related making it the leading cause of injury for this population in western societies [3]. Risk factors such as increasing level of competition, training duration and intensity [4] were consistently identified across 15 different sporting disciplines [5] and deemed responsible for the high injury rates. However, despite increasing research and knowledge of injury mechanisms (e.g. anterior cruciate ligament injury [6]), risk factors and recovery methods, injury rates remain high in both the youth [5] and professional [7] ranks.

There is a general agreement among sport professionals that injury has an unfavorable impact on the sport they are involved in. In a survey that collaborated with 72 professionals within soccer, 77% believe that injury has a negative impact on their teams overall performance [8]. Additionally, 98% of soccer professionals share the belief that evidence based injury prevention exercises should be applied to their regular training routine [8]. Injury prevention programs (IPPs) such as the FIFA 11+ [9], [10], the PEP Program [11] and Sportsmetrics [12] have been previously designed and proven effective in preventing sports-related injuries. However, long-term implementation of IPPs is often problematic as proving effectiveness against injury is insufficient for coaches and athletes to convert from their conventional routine [13].

Additionally, modified versions of evidence based programs are partially implemented to add variation, progression and individualization as well as to align with specific training formats and goals [8]. In the survey, the FIFA 11+ was implemented in their original (12% of sessions) and modified version (28% of sessions) [8]. This could be attributed to the fact compliance to an IPP is highly dependent on the exercises being enjoyable, practicable, progressive and result in physical benefits for their athletes [10,14], which often manifests itself in the form of exercises that pertain directly to the given sport e.g. sports specific.

Specificity is recognized by coaches as a key principle of training that result in training adaptations and is therefore, frequently carried out in sessions [15]. If it can be revealed that specific IPPs lead to performance improvement in comparison to other types of IPPs, then coaches would be assured that specificity should remain an integral part of their training time with their athletes.

A previous systematic review conducted by the present authors aimed to determine whether specificity of an IPP affects the injury rates of young athletes [16]. One main finding was that there is currently a dearth of research using sports specific IPPs (n = 2) as per the authors´ definition, thus making practical recommendations challenging. Due to this, a new perspective that hasn’t previously been addressed is: Does additional injury prevention training culminate in performance improvement in athletes? If so, then does the specificity of the program make a difference in enhancing crucial performance parameters? Or, could it be the case that programs with general training exercises attribute to this improvement and thus resulting in a one size fits all type approach. There are IPPs that integrate tasks that are more sports-specific e. g. using cutting maneuvers and jumping/landing exercises [17] [18]. Conversely, other IPPs aim to develop general fitness abilities indirectly related to the athlete’s sport e.g. core stability exercises such as squats and side planks [19]. It remains unclear, which of these program types (sports specific or general IPPs) have the highest evidence-based justification for implementation regarding the positive effects on critical performance indices. Therefore, the aim of the present systematic review was to evaluate the effectiveness of evidence based IPPs on performance in young athletes.

## Methods

### Data sources

A systematic search of the literature, in compliance with the preferred reporting items for Systematic Reviews and Meta-Analyses [20] was conducted.

Relevant articles were identified via PubMed and Web of Science and were systematically searched between Jan 2006 until May 2019. The search terms used were “*athletes”* AND “*injury prevention” OR “exercise program*”. Two authors (HM; AP) performed the literature search independently, with disagreements resolved by consensus and further consultation from a third author (JM) was sought if necessary. The search process entailed removing duplicates, screening titles, abstracts and eligible full texts. Additionally, reference lists of excluded systematic reviews, meta-analyses and reviews were manually searched to identify studies of relevance.

### Eligibility criteria

Inclusion criteria: with the aim of evaluating the effectiveness on performance.

1. Individual and team sport athletes aged 11–45 years who had to have either participated in an IPP or have kept their usual training routine/standardized protocol.
2. The design of the study was restricted to randomized controlled trails (RCT) and non-randomized control trial (NRS).
3. Included at least one of the following outcome measures; a) dynamic balance (e.g. star excursion balance test), b) static balance (e.g. centre of pressure), c) power (e.g. jump height), d) strength (e.g. isokinetic strength at different angle velocities), e) endurance (e.g. repetitions per minute) or f) speed/agility (e.g. timed short distance tests) (S3 Table)
4. Had at least two measurement time points; before and after the IPP. In the event of multiple post intervention assessments, the measurement immediately post-intervention was used.

There were no limits placed on athletic level, sex or duration of the intervention. Passive IPPs, among others based on stretching, were considered irrelevant and excluded.

### Data extraction

The following data was extracted from each eligible full text: the first author’s last name, publication year, study design, country, follow-up period, study duration, type of sport, exposure data, subject information (sample size, dropout rate, sex and age) and intervention (name, description, type, dose, frequency, compliance, effects and categorization according to authors’ definition).

### Data analysis

The IPPs’ were categorized into three sections: 1) general, 2) mixed and 3) sports-specific. Each exercise that comprises an IPP was determined by the authors and added to a category which most aptly describes them; **category a):** The exercise directly relates to the movement, task or skill performed in the athletes’ respected sports (e.g. a jumping motion in a program for basketball players) or **category b)** The exercise focuses on developing general physical abilities that do not directly pertain to the movement, task or skill performed in their sport. Sports-specific IPPs were defined as IPPs which comprise primarily (i.e. >80%) of exercises from category a and general IPPs were defined as programs that consisted of exercises (i.e. >80%) from that described in category b. In cases where the components did not show a clear direction, i.e. either 21–79% not related or related to the sport, were categorized as a mixed IPP. For clarity, this is illustrated in the already published first part of this review assessing the effects of IPPs on injury rates [16]. This review focuses on the various IPPs effect on performance. Table 1 displays a summary of the main characteristics in each IPP type (e.g. intervention/control group, age range, sport type and target extremity). Table 2 identifies the tests used to assess the performance parameters in the included studies whereas Table 3 displays the overall effectiveness of the IPPs on various sports performance parameters. For clarity, the effectiveness of a given IPP is determined whether within each study the outcome measurement is significantly improved versus control e.g. In a study by Hermassi et al. [21], they used what would be defined as a sports specific IPP and assessed whether that increased throwing velocity [m.s^-1^], which is a measurement of power. It could then be determined whether the intervention group improved this measure significantly against the control group.

**Table 1:** Study characteristics of included studies.

IPP Type	General	Mixed	Sports Specific
Study No.	11	8	9
N Intervention	185	179	119
N Control	167	179	100
Age	12–25	10–21	14–27
Intervention type	9 Training programs [23] [24] [25] [26] [19] [27] [28] [29] [30] 2 Warm-up [31] [32]	3 Training programs [33] [34] [35] 5 Warm-up [36] [37] [14] [18] [38]	9 Training programs [39] [40] [41] [42] [43] [21] [44] [45] [46]
Target extremity	8 Lower extremity [23] [24] [25] [26] [19] [27] [28] [30] 1 Upper extremity [29]	8 Lower extremity [33] [34] [35] [36] [37] [38] [14] [18]	7 Lower extremity [39] [40] [42] [43] [21] [45] [46] 2 Upper extremity [41] [44]
Sport	6 Football [23] [24] [26] [32] [27] [30] 1 Handball [25]	7 Football [10] [14] [18] [47] [35] [17] [33] 1 Basketball [34]	3 Basketball [39] [40] [46] 1 Handball [25] [21] 1 Football [43] 1 Hockey [42] 1 Track Sprinting [45] 1 Tennis [44],

**Table 2:** Outcome measures used in the five performance categories.

Dynamic Balance	SEBT (x3) [24] [32] [48]	SEBT [17]	SEBT [39]
Static Balance	One legged stand time [19] Time to stabilization, BESS, COP_sway_ [31]	Stability [18] COP [34] Single leg balance [17]	COP, Centre of gravity control [49]
Power	CMJ (x8) [30] [25] [28] [29] [27] [26] [23] [32], Squat jump (x4) [23] [25] [30] [26], Vertical Jump [19] 5-jump test, Drop jump [30] 20 & 40cm drop jump [28], Kicking performance [27], Absolute power, Maximal force [23], Triple hop distance [32]	CMJ (x3) [14] [33] [36] Average kicking distance, Vertical jump height [35] 3 Step jump [14], Vertical drop jump, distance kick [36] Single legged triple hop [17]	Ball throwing velocity [21] Running based anaerobic sprint test- Max. and mean power [40] Standing throw, Jumping throw, Throw with run [25]
Strength/ Endurance	Push up, Sit up, Grip strength [19], Bench press, Full squat, [29] Trunk muscular strength [27] Hip extension/flexion torque [26] Maximal lower limb load [25]	Squatting, Stepping, Lunging, Leg raising, Push up [18], Concentric/eccentric knee flexors/extensors peak torque [47] Core stability [14] Isokinetic leg strength, Isometric leg strength [36]	Concentric and eccentric external/internal rotation [21] Concentric/Eccentric shoulder peak torque/Total work [44] Pull over, Bench press [25]
	Yo-Yo test, Maximal aerobic speed [30] 2.4km [28]	Bar jumps [17]	Running based anaerobic sprint test- fatigue [40]
Speed/Agility	20m sprint (x4) [30] [26] [28] [32], 10m sprint (x3) [26] [30] [32], T-test (x2) [30] [27], Side Step [19] 10m, 30m sprint [30], 15m sprint [26] Sprint velocity [23] Illinois agility test [32]	Agility, 20m sprint [14] 40m sprint, Shuttle run [36] 9.1m,18.2m,27.3m,36.6m sprint, Illinois and pro agility test [33]	T-test [40] [46] Total/fastest/slowest/mean sprint time [42] 7.32m 10m Shuttle and Timed circuit test [43]

**BESS**, Balance Error Scoring System; **CMJ**, Countermovement jump; **m**, meters **min.**, minutes; **COP**, Centre of Pressure; **SEBT**, Star Excursion Balance Test; **s**, seconds

**Table 3:** A summary of the effectiveness of each outcome measure in each of the 3 IPP types.

IPP Type	General		Mixed		Sports Specific	
	Eff.	NE	Eff.	NE	Eff.	NE
**Balance**	*4*	*3*	*4*	*1*	*2*	*1*
**Effectiveness**	*57%*		*80%*		*66%*	
**Power**	7	14	*4*	*5*	*5*	*1*
**Effectiveness**	*33%*		*44%*		*83%*	
**Strength**	*4*	*4*	*2*	*8*	*6*	*2*
**Effectiveness**	*50%*		*20%*		*75%*	
**Endurance**	*3*	*0*	*1*	*0*	*1*	*0*
**Effectiveness**	*100%*		*100%*		*100%*	
**Speed/Agility**	*4*	*10*	*3*	*7*	*8*	*5*
**Effectiveness**	*29%*		*30%*		*62%*	

**Eff.**: effective; **NE:** not effective

### Risk of Bias assessment

The internal validity of the RCTs was assessed using the Cochrane Collaborations’ risk of bias assessment tool [22]. Independently, the two authors (HM; AP) examined the studies of interest for the following sources of bias: selection (sequence generation and allocation concealment), performance (blinding of participants/personnel), detection (blinding outcome assessors), attrition (incomplete outcome data), reporting (selective reporting) and other potential bias.

## Results

**Fig 1:**
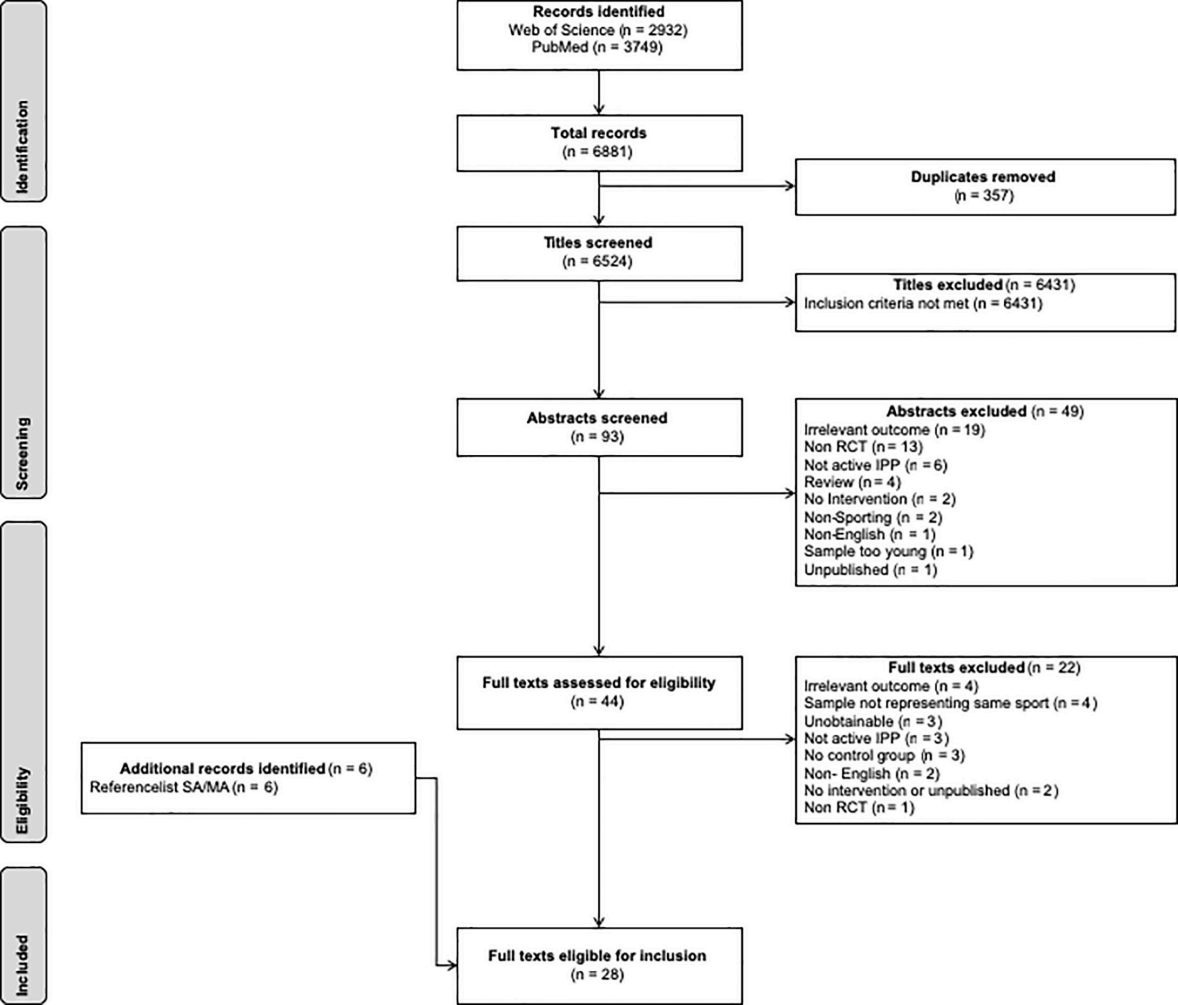
Flowchart for screening and selection of studies according to PRISMA.

The initial search identified 6881 potentially relevant studies (Fig 1). Duplicates, reports, general review articles, current concepts, commentaries, systematic reviews and meta-analyses as well as studies that did not match the inclusion criteria were excluded following screening of titles and abstracts. Forty-four studies remained for full text screening and of which, 22 were found to be ineligible. Additional evidence was retrieved via reference lists of previously excluded reviews which unearthed a further 6 suitable articles. Consequently, 28 studies met the inclusion criteria for the investigation.

Table 1 displays a summary of the study characteristics. Twenty one studies applied a training intervention in addition to regular training and 7 studies used a warm-up intervention in place of the usual warm-up routine [14] [18] [31] [32] [36] [37] [38]. Stated compliance in the 10 IPPs ranged from 24–100% [50] [26] [27] [48] [17] [18] [10] [14] [33] [21] whereas 18 were undefined [23] [24] [25] [19] [28] [29] [30] [47] [34] [35] [39] [40] [41] [42] [51] [49] [44] [46].

The S1–S3 Tables give a comprehensive overview of the interventions, study characteristics and results. In the interest of coherence and readability, the programs will be collectivized into first either a) General, b) Mixed or C) Sports-specific. No specific mention will be given to the type of intervention (e.g. strength program, plyometrics etc.) but references will be giving for every reporting of a result.

### General IPPs

Of the 11 studies comprising of general IPPs, 3 assessed dynamic balance [32] [31] [24], 2 static balance [31] [19], 4 strength (10 overall measures) [19] [29] [27] [26], 8 power (20 overall measures) [30] [28] [29] [27] [26] [23] [32], 2 endurance [30] [28] and 7 speed/agility (14 overall measures) [19] [30] [28] [27] [26] [23] [32] as a function of performance. The IPPs ranged from 1–29 different exercises lasting between 10-90min. and were applied 2 to 5 times per week for the duration of 4 to 13 weeks.

#### Balance

Three studies measured dynamic balance as expressed by the Star Excursion Balance Test (SEBT). Two of which (warm up programs) show no improvement in SEBT score [32] [48]. The same general neuromuscular warm-up program assessed 3 measures of static balance and only the Balance Error Scoring System (BESS) improved whilst Time-to-Stabilization and Centre of Pressure (COP) sway were unaffected relative to control [31]. In active interventions, SEBT was improved in 3 out of 6 measures in one study [24] whilst another found that closed eyes one legged stand significantly improved compared to control [19].

#### Power

There were 17 measures of various jump types across 8 active IPPs [19] [30] [28] [29] [27], [26] [23] and a single warm up program [32]. Six jump measures were increased compared to control and 11 did not differ significantly. The other power measure that improved was maximal force [23]. Kicking performance [27] and absolute power [23] failed to show a significant increase versus control.

#### Strength

Bench press, full squat weight, hip extension torque (although not hip flexion torque) and maximal lower limb load were significantly improved following active IPPs [29] [26] [25]. Conversely, push-up/sit-up number and grip strength [19] remained unaffected. A general warm up IPP led to no significant changes in trunk muscular strength [27].

#### Endurance

Maximal aerobic speed / yo-yo test and a 2.4km time trial increased significant compared to control group following 2 active strength protocols [30], [28].

#### Speed/Agility

Five studies measured 9 types of sprint speed (e.g. 20m), only 3 improved following 2 different active strength IPPs [30] [28]. Highest sprint velocity was significantly improved following a warm up plyometric protocol [23].

For tests of agility, 4 measures in 4 different studies failed to yield significant improvement. Side Step [19], [30] [27] [32] failed to improve compared to control.

### Mixed IPPs

Of the 8 studies comprising of mixed IPPs, a single study assessed dynamic balance [17] as an aspect of performance, 3 static balance [17] [34] [18], 5 power (9 overall measures) [35] [14] [36] [17] [33], 4 strength (10 overall measures) [18] [38] [14] [36], 3 speed/agility (10 overall measures) [14] [36] [33] and 1 endurance [17]. The IPPs comprised 12–41 different exercises lasting between 10-120min. and were applied 2–5 times per week for the duration of 4 weeks-20 months.

#### Balance

The FIFA 11+ is a neuromuscular warm-up program and is commonly applied in studies attempting to determine its efficacy, one study [17] found a significant improvement in 2 from 6 possible angles in the dynamic SEBT following FIFA 11+. As for static balance, the single legged eyes closed balance test in both legs [17] was significantly improved compared to the control group. Another study that applied the FIFA 11+ program failed to show improvement in functional stability amongst young male footballers [18]. A 20 week neuromuscular warm-up showed significant improvement COP, particularly in the dominant limb [34].

#### Power

CMJ was measured in 3 separate studies [33] [14] [36]. Following the PEP program and the F-MARC 11, this measure was not improved [33] [36] although a study implementing the same program (F-MARC 11) found significant improvement versus control [14]. Football kicking distance, vertical jump height and improved post plyometric training [35]. The F-MARC 11 program did not result in significant improvement in vertical drop jump [36] or kick distance [36] and the updated version of the program (FIFA 11+) did not significantly increase single legged triple hop distance [17].

#### Strength

A study that measured knee extensor torque found a concentric improvement without much effect on the knee flexors when working eccentrically following a specific knee IPP [47]. Amongst three studies, the F-MARC 11 or the FIFA 11+ program resulted in no difference in core stability [14] isokinetic/isometric leg strength [36], squatting, stepping, lunging, leg raising or push-ups [18].

#### Endurance

The only measure of endurance that was found for mixed programs was bar jumps which was assessed before and after the FIFA 11+ [17] and was significantly improved.

#### Speed/Agility

Sprint times for 20m [14] 27.3m and 36.6m [33] in 2 separate studies were significantly improved post IPP. Agility [14] 40m sprint, Shuttle run [36] 9.1m,18.2m sprint, Illinois and pro agility test [33] were all unaffected by the F-MARC 11 [14] [36] or the PEP program [33].

### Sport-specific IPPs

Of the 9 studies comprising of sports specific IPPs, 1 assessed dynamic balance [39], 1 static balance (2 overall measures) [45], 3 power (7 overall measures) [21] [40] [25], 3 strength (8 overall measures) [21] [44] [25], 4 speed/agility (9 overall measures) [40] [42] [43] [46] and 1 endurance [40] as a function of performance. The IPPs ranged from 1–20 different exercises lasting between 15-60min. and were applied 2 to 5 times per week for the duration of 3 to 8 weeks.

#### Balance

Following plyometric training, the SEBT improved in 6 out of 8 directions [39], The progressive sprinter-specific proprioception training program found significantly better stability with eyes open in the medial-lateral plane in the experimental group. Furthermore, gravity control was measured and did not yield in statistical significant differences, although differences on the right side and the back were reported [45].

#### Power

Strength training led to no significant increase in ball throwing velocity compared to control [21]. Conversely, mid-season resistance training improved the standing, jumping and running throw in handball athletes [25]. Sports specific plyometric training led to significant mean and maximum (w) anaerobic sprinting power gains [40].

#### Strength

Eccentric and concentric shoulder peak torque improved during a strength training program albeit not compared to control. The total work of the internal/external rotators did significantly improve in the same study [44]. Another strength training IPP only found concentric and eccentric improvements of the external/internal rotators of the non-dominant arm [21]. Sports-specific resistance training resulted in increased strength (kg) in pull overs and bench press [25].

#### Endurance

Running based anaerobic sprint test- fatigue improved following plyometric training in a single study investigating endurance [40].

#### Speed/Agility

Two studies implementing plyometric training and dynamic balance training improved post-protocol t-test scores [40] [46]. A significant between group interaction effect was observed at total, fastest, slowest and mean linear sprint time as well as shuttle sprints with and without a ball after completing sprint training [42], although no difference was observed during the slalom runs. A group x time effect was observed following soccer specific training but no significant difference in the 7.32m, 10m Shuttle and Timed circuit test were apparent [43].

### Risk of bias assessment

**Fig 2:**
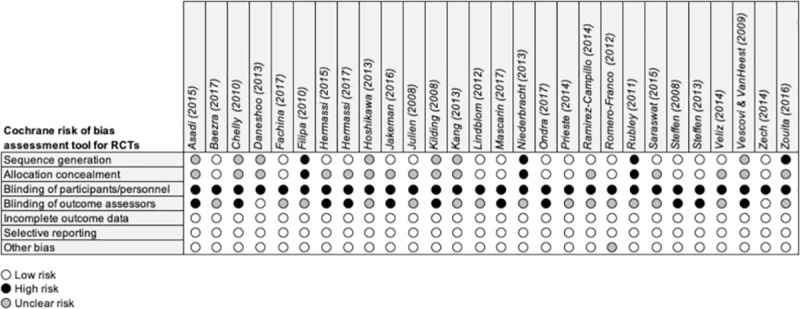
Risk of bias assessment of included RCTs.

The results of the methodological quality assessment across all included RCTs is presented in Fig 2.

## Discussion

The present systematic review presented the effectiveness of general versus sports-specific IPPs and their role on enhancing key indices of athletic performance. The current consensus amongst coaches is that the highest performance benefit can only be achieved through implementation of a sports-specific IPPs. Although not entirely in line of the presented evidence, there is no apparent necessity to deviate from this existing perception as the results are largely positive in favor of specificity to the sport concerned.

The first part of the current research attempted to define the definition of sports-specific. This has been discussed in a previous review [16]. The intention was to define a sports specific program as one that consisted of maneuvers whereby >80% are used in the sport itself. e.g. starting, stopping, twisting, turning, running, jumping, landing, shuffling, pushing, pulling, hitting, throwing, catching, hopping, accelerating, decelerating, sliding, blocking. Isolated actions such as planks, bench press and squats do not directly correspond to the movement pattern involved in any sport and would be considered as general movements. The research attempted to address this question and to identify possible avenues for future research.

As summarized in Table 3, general programs arguably show a modest effect (29–57%) on all performance outcomes except for endurance (100%). Mixed interventions resulted in a relatively low success rate in measurements of strength (20%), power (44%) and speed (30%) whereas the sports-specific programs shown significant improvement in every measurement of endurance, 75% of strength measurements and power, balance and speed/agility were largely positive in their effectiveness (63–66%).

The sports-specific IPPs generally show a more positive relationship in terms of effectiveness in power, strength and speed/agility when compared to the other types. In a sporting environment, being able to perform repeated explosive movements such as rapid acceleration/deceleration (i.e. power) and changing direction (i.e. agility) is critical to success and injury risk reduction. Power production is stated to be influenced by motor unit synchronization, neuronal adaptation [25] and increases in neural activation [30]. Whereas agility is linked to ATP production [40], ATP resynthesis [42] and thereby reducing the time required for voluntary muscle activation. This reduction would manifest itself in faster changes in movement and therefore more agility. In this regard, the speed of movement should provide the neuromuscular stimulation to mimic that performed in the explosive movements of match play. This stimulus often comes in the form of sports-specific exercises whilst a lot of mixed and general programs fail to replicate this [32] [19] [36] [14] [27] [29] [26] [17] [33] [30] perhaps thereby explaining the discrepancy in the results. A few notable exceptions is that a few general programs did see increases in at least one measure of power following strength training programs [30] [25] [28] so it would appear IPPs that progress their exercises (by either increasing the resistance or degree of difficulty) or have movements in the program that directly replicate the outcome of the assessed movement (e.g. in the case of Chelly et al. (2010), the IPP consisted of hurdle and drop jumps and used CMJ and squat jumps as the performance outcome) lead to some improvement in power. Additionally, there were incidences of improved sprint speeds [14] [33] and jumps [14] [35] following mixed IPPs and it was suggested in the literature that plyometric exercises could have prompted these enhancements [14].

If we examine strength acquisition, all three sports specific IPPs improved versus control [25] [21] [44]. The unifying factor of these IPPs is that resistance level was progressively increased during the time period to adapt to the athletes’ current strength level. In one of these studies, sports specific strengthening resulted in a tangible performance improvement in handball players [25], it was observed that all throwing types were significantly faster (m.s^-1^) than their control counterparts. This type of sports specific IPP may appeal to both coaches and players alike. If for example, a team has been suffering with multiple injuries of the same nature e.g. there is a high incidence of hamstring strains in football [52], general functional strengthening may be appropriate to either add to regular training or be implemented as an off season IPP as improvements are observed [27] [19], but in regards to warm-up programs of mixed exercises (e.g. the FIFA 11 and 11+), there was less significant improvements in strength [18] [47] [14] [36]. This effect is largely attributed to the program being short in its duration of 15-20mins and not providing the necessary stimulus for strength acquisition.

When examining the influence of IPP content on balance, certainly a necessary stimulus should replicate the unpredictable high-speed environment of sport, but simultaneously, having a certain level of general thigh, hip, ankle and core strength is also an important factor [17]. In this regard, a program of mixed exercises for balance improvement may be the most efficacious [17] [34]. Interestingly, in the mixed IPPs, there were two studies that investigated dynamic or static balance following FIFA 11+ (which is also considered a warm up program) [17] [18] with the only difference one was lasting longer (16 [17] weeks compared to 6 [18]) and was coach focused (i.e. supervised and corrected). The same authors uniquely tested for and indeed found a dose-response relationship between the volume of FIFA 11+ exercises performed and the magnitude of balance performance. On this basis, dosage and compliance should be factored in to any future investigations before assessing the true effectiveness of the program itself.

A prior review suggested that regardless of low volume, the FIFA 11+ warm-up is enough to improve balance [53]. Although the FIFA 11+ was originally intended for footballers, there is no reason why these abilities cannot be transferable to other sports that replicate some of the movement patterns e.g. hockey, basketball, rugby and handball. There is a general lack of studies that measured endurance, but the ones that do show significant improvement versus the control group regardless of IPP content [30] [28] [17] [40]. Future research should perhaps consider endurance more intently as an outcome measure after IPP intervention.

### Recommendations

The authors can reasonably make some creditable recommendations based on the presented results. For future researchers, it is crucial that other factors such as compliance, age and standard of the athletes, time of season, facility access and execution of exercises are well considered to reveal the true effectiveness and therefore clinical relevance of IPPs. A dose-response relationship should be investigated which has previously been found over the course of 4 months for key performance indicators [17]. For coaches, particularly of young athletes, IPPs should be applied to prevent injuries [16]. However, do coaches need to consider the IPP type to gain an additional benefit to performance? It appears that IPPs that focus on general exercises do not provide an additional performance benefit and it is therefore recommended that sports specific IPPs are desirable.

In accordance with the TRIPP model, proving an injury reducing effect of IPPs (e.g. the FIFA 11+ [54]) is often insufficient for coaches and athletes alike to yield implementation [13]. Over the course of a season, providing tangible performance benefits (short and long-term) may increase the likelihood of real-world implementation. A further recommendation is that professionals with less time constraints or recreational athletes in the off season may benefit from longer duration (>30min) neuromuscular training that has been shown to improve strength [26] [29] [44] [21] [25], balance [19] [39], power [30] [28] [29] [21] [40] speed/agility [30] [28] [40] and endurance [30] [28] [40] in several studies regardless of IPP content.

### Risk of bias assessment

The overall quality of the included RCTs was considered moderate. Sequence generation and allocation concealment in these sorts of studies are inherently difficult. Moreover, blinding of participants as well as personnel for exercise interventions is not feasible. Moreover compliance of such IPPs mostly rely on self-reports; therefore, all trials showed high risk of performance and detection bias. Nevertheless, the overall quality could have been underestimated by non-transparent reporting. Accordingly, consensus statements such as CONSORT [55] should be followed across studies.

### Study limitations

It could be argued that the initial search strategy lacked robustness, with only two databases searched and six articles retrieved during the manual screening of reference lists from systematic reviews. The largest limitation is the considerable number of variables of an IPP and its implementation into a study design (e.g. the repetitions and execution of exercise, the compliance rate). The sizeable number of outcome measures that assess performance lead to further complications when attempting to determine the overall IPP effectiveness. Furthermore, no standard tests exist that truly correlates with the performance and the internal validity of performance studies may thus be constrained.

### Future research

In order to make recommendations regarding the effectiveness of sports-specific IPPs on athletic performance (short or long-term), future investigation need to compare effects of sports-specific versus general IPPs. To allow further comparisons, outcome measures would ideally be similar across investigations. Moreover, to control for adequate compliance/application of the IPPs, future studies have to invest more resources in following up on athletes and coaches/training staff without rising concern about potential motivational bias.

## Conclusion

There are contradictory findings regarding the effect that neuromuscular programmes may have in improving physical performance. A large majority of sports-specific IPPs did improve outome measure that the researchers found to be of use so in this regard, additional sports-specific training does indeed benefit performance. It cannot be unstated, however, that key performance improvements are potentially affected by a variety of other factors (e.g. compliance, frequency, intensity, technical execution) so it is difficult to assess the magnitude of influence that specificity to the sport actually has.

## Supporting information

S1 Table Summary of intervention programs used in the included studies (alphabetical order by program). Level**: E, professional/elite/highest level; AM, amateur; d, days; min., minutes; mo., months; N/D, not described; reps, repetitions; S, season; SE, session(s); VB, volleyball; wk., weeks; yrs., years.
Values presented as mean ± standard deviation if not otherwise stated.
(DOCX)

S2 Table Exercise interventions applied by included studies (alphabetical order). ACL, anterior cruciate ligament; BB, basketball; CPL, compliance; EX, exercise(s); KLIP Program, Knee Ligament Injury Prevention Program; LE, lower extremity; min., minutes; N/D, not described; OSTRC, Oslo Sports Trauma Research Center; PEP Program, Prevent injury and Enhance Performance Program; Pt., part/phase; reps, repetitions; s, seconds; SE, session(s); UE, upper extremity; VB, volleyball; wk., week(s); yrs., years.
(DOCX)

S3 Table Results and conclusion of included studies (alphabetical order). “Statistically significant difference within groups (pre- post-test). * Statistically significant difference between intervention and control group (pre- post-test).
5JT, 5 jump test; BAL, balance training group; BESS, Balance error scoring system; CG control, general gravity center control; CMJ, counter movement jump; COP, center of pressure; DEO, distance covered by the center of pressure with eyes open; DJ, drop jump; ECC, Eccentric strengthening exercises; ER, external rotation; IR, internal rotation; MAS, maximal aerobic speed; MSFT, multi-stage fitness test; PLYO, plyometric training group; RAST, running-based anaerobic sprint test; RJ; rebound jump,; RombergD, Romberg index about distance; RombergS, Romberg index about surface; RombergSp, Romberg index about speed; SE, session(s); SEBT, Star Excursion Balance Test; SEO, surface covered by the center of pressure with eyes open; SLH, single leg hop; SLTH, single leg triple crossover hop; SLS, single leg squat; SpEO, speed of center of pressure movement with eyes open; THDT, Triple hop distance; TTS, time to stabilization; UNS, unstable surface exercises; VDJ, vertical drop jump; VO2max, maximal oxygen uptake; XEO, mean position center of pressure in the medial-lateral plane with eyes open; YEO, mean position center of pressure in the anterior-posterior plane with eyes open; YYIRTL1, Yo-Yo intermittent recovery test level 1; Values presented as mean ± standard deviation or Δ% or if not otherwise stated.
(DOCX)

S1 File PRISMA checklist. (PDF)

S2 File Search strategy. (XLSX)
