# Supplementary material for: General versus sports-specific injury prevention programs in athletes: A systematic review on the effects on performance
Source: PLoS One. 2019 Aug 29;14(8):e0221346. doi: 10.1371/journal.pone.0221346 (PMC6715272; doi:10.1371/journal.pone.0221346)
Supplement: S1 Table — Level**: E, professional/elite/highest level; AM, amateur; d, days; min., minutes; mo., months; N/D, not described; reps, repetitions; S, season; SE, session(s); VB, volleyball; wk., weeks; yrs., years. Values presented as mean ± standard deviation if not otherwise stated. (DOCX) [file pone.0221346.s001.docx]

**Table S1.** *Study characteristics of included studies (alphabetical order)*

| **Author (Year)** **Design** **Country** | **Population (Level**)** | **Subjects** | | | | | | | | | | | **Intervention** | | | |
| --- | --- | --- | --- | --- | --- | --- | --- | --- | --- | --- | --- | --- | --- | --- | --- | --- |
|  |  | **Intervention** | | | | | | **Control** | | | | | **Intervention** | | **Control** | |
|  |  | **N** | | **Sex** | | **Age in yrs** | | **N** | **Sex** | | **Age in yrs** | |  |  |  |  |
| **General** | | | | | | | | | | | | | | | | |
| *Chelly (2010)*  Canada | Football  (AM) | 12 | | ♀ | | 19 ± 0.7 | | 11 | ♀ | | 19 ± 0.7 | | *Lower Limb Plyometric Training*  [8-wk./2 x wk./ 30min.] | | Followed their normal training routine. | |
| *Filipa (2010)*  *USA* | Football  (AM) | 13 | | ♂ | | 15.4 ± 1.5 | | 13 | ♂ | | 14.7 ± 0.8 | | *Neuromuscular Program*  [8-wk./ 2 x wk./ ~ 45min.] | | Followed their normal training routine. | |
| Hermassi (2017)  *Germany* | Handball  (AM) | 12 | | ♀ | | 18.9 ± 0.2 | | 10 | ♀ | | 18.9 ± 0.6 | | *Strength Training Program*  [10-wk./ 2 x wk./ ~ 20min.] | | Followed their normal training routine. | |
| *Hoshikawa (2013)*  *Japan* | Football  (AM) | 16 | | ♀ | | 12.3 – 13.1 | | 12 | ♀ | | 12.3 – 13.1 | | *Stabilization Training Program*  [6-mo./ 4 x wk.] | | Soccer training 5 days per wk. (2 hours) with one game on the weekend. | |
| *Kang (2013)*  South Korea | Weightlifting  (AM) | 16 | | ♀ | | 15.5 | | 16 | ♀ | | 15.5 | | *Balance Training Program*  [8-wk.] | | N/R | |
| *Lindblom (2012)*  Sweden | Soccer  (AM) | 23 | | ♂ | | 14.2 ± 0.7 | | 18 | ♂ | | 14.2 ± 1.1 | | *Neuromuscular Warm-Up Program*  [11-wk./2 x wk. /15min] | | Used standardized warm-up. | |
| *Prieske*  *(2014)*  Germany | Football  (E) | 19 | ♀ | | 16.6 ± 1 | | 20 | | | ♀ | | 16.6 ± 1.1 | | *Core Strengthening Training Program*  [9-wk./ 2-3 x wk.] | | Same exercises on a stable surface. |
| *Ramirez- Campillo (2014)*  *Spain* | Running  (AM) | 17 | ♀ 9  ♂ 8 | | N/R | | 15 | | | ♀10  ♂ 5 | | N/R | | *Explosive Strength Training*  [6-wk./ 2 x wk./ 30min.] | | Kept their usual volume of running endurance training. |
| *Ramos Velez (2014)*  *Spain* | Waterpolo  (E) | 16 | ♀ | | 20.43 ± 5.09 | | 11 | | | ♀ | | 20.43 ± 5.09 | | *Strength and High Intensity Program*  [18-wk./ 2 x wk./ 30-45min.] | | Followed their normal water training routine. |
| *Zech (2014)*  Germany | Field hockey  (AM) | 15 | ♀ | | 15.7 ± 3.9 | | 15 | | | ♀ | | 14.1 ± 1.4 | | *Neuromuscular Warm-Up Program* [10-wk./2 x wk./ 20min.] | | Followed their normal training routine. |
| Zouita (2016)  Tunisia | Football  (E) | 26 | ♀ | | 13 – 14 | | 26 | | | ♀ | | 13 – 14 | | *Strength training*  [12-wk./ 3 x wk./ 90min.] | | Followed their normal training routine. |
| **Mixed** | | | | | | | | | | | | | | | | |
| *Baeza (2017)*  *Chile* | Football  (AM) | 11 | | ♀ | | 13.45 ± 0.7 | | 11 | ♀ | | 13.36 ± 0.5 | | *FIFA 11+* [6-wk./3 x wk./20min.] | | Normal routine: running exercises, stretching and joint movement.  [6-wk./3 x wk./20min.] | |
| *Daneshjoo (2013)*  *Malaysia* | Football  (E) | 12 | | ♀ | | 18.9 ± 1.4 | | 12 | ♀ | | 18.9 ± 1.4 | | *FIFA 11+*  [8-wk./24 SE] | | Followed their normal training routine. | |
|  |  | 12 | | ♀ | | 18.9 ± 1.4 | |  |  |  |  |  | *Harmoknee*  [8-wk./24 SE] | |  |  |
| *Kilding (2008)*  New Zealand | Football  (AM) | 12 | | ♀ | | 10.4 ± 1.4 | | 12 | ♀ | | 10.4 ± 1.4 | | *FIFA 11*  [6-wk./5 x wk./ 15min.] | | Followed their normal training routine. | |

*Continued*

*Continued*

| **Author (Year)** **Design** **Country** | **Population (Level**)** | **Subjects** | | | | | | | | | | | **Intervention** | | | |
| --- | --- | --- | --- | --- | --- | --- | --- | --- | --- | --- | --- | --- | --- | --- | --- | --- |
|  |  | **Intervention** | | | | | | **Control** | | | | | **Intervention** | | **Control** | |
|  |  | **N** | | **Sex** | | **Age in yrs** | | **N** | **Sex** | | **Age in yrs** | |  |  |  |  |
| **Mixed** | | | | | | | | | | | | | | | | |
| *Ondra (2017)*  *Czech Republic* | Basketball  (AM) | 10 | | ♀ | | 17.3 ± 1.3 | | 11 | ♀ | | 16.5 ± 1.8 | | *Neuromuscular Training* Program  [20-wk./3 x wk./20min.] | | Followed their normal training routine. | |
| *Rubley (2011)*  *USA* | Football  (AM) | 10 | | ♂ | | 13.4 ± 0.5 | | 6 | ♂ | | 13.4 ± 0.5 | | *Plyometric Training*  [14-wk./ 2-3 x wk.] | | Followed their normal in season soccer training routine. | |
| *Steffen (2008)*  Norway | Football  (AM) | 18 | | ♂ | | 17.1 ± 0.8 | | 16 | ♂ | | 17.1 ± 0.8 | | *F-MARC 11*  [10-wk./3 x wk. / 15min.] | | Followed their normal training routine. | |
| *Steffen (2013)*  Norway | Football  (AM) | 78 | | ♂ | | 13 – 18 | | 80 | ♂ | | 13 – 18 | | *FIFA 11+*  [pS (2 mo.)/ 2-3 x wk./ 20 min.] | | Coaches provided with online access to training program, no expert intervention. | |
| *Vescovi & VanHeest (2009)*  Canada | Football  (AM) | 16 | | ♂ | | 15.7 ± 1.2 | | 15 | ♂ | | 16.8 ± 0.4 | | *PEP Program* [12 wk./3 x wk./15min.] | | Followed their normal training routine. | |
| **Sports Specific** | | | | | | | | | | | | | | | | |
| *Asadi (2015)*  *Iran* | Basketball  (AM) | 8 | ♀ | | 20.1 ± 0.8 | | 8 | | | ♀ | | 20.5 ± 0.3 | | *Plyometric program*  [6-wk./ 2 x wk./ 60min.] | | Followed their normal basketball training routine. |
| *Fachina (2017)*  Brazil | Basketball  (AM) | 18 | ♀ | | 15.2 ± 2.7 | | 21 | | | ♀ | | 16.4 ± 2.6 | | *Plyometric Training*  [8-wk./ 3 x wk. ~60min.] | | Followed their normal training routine. |
| *Hermassi (2015)*  Tunisia | Handball  (E) | 12 | ♀ | | 18.7 ± 0.5 | | 10 | | | ♀ | | 18.4 ± 0.5 | | *In-Season Resistance Training*  [8-wk./ 3 x wk.] | | Did not take part in any supplementary training. |
|  |  | 12 | ♀ | | 18.5 ± 0.5 | |  |  |  |  |  |  |  | *In-Season Resistance Training (Resistance Throwing)*  [8-wk./ 3 x wk.] | |  |
| *Jakeman (2016)*  *United Kingdom* | Hockey  (E) | 7 | ♂ | | 18 – 26 | | 7 | | | ♂ | | 18 – 26 | | *High Intensity Training*  [4-wk./ 2 x wk.] | | Followed their normal training routine. |
| *Julien (2008)*  France | Football  (E) | 8 | ♀ | | 19.9 ± 3.4 | | 9 | | | ♀ | | 18.9 ± 1.7 | | *Co-ordination training*  [3-wk./ 5 x wk.] | | The reference group performed technical work. |
|  |  | 9 | ♀ | | 18.4 ± 1.6 | |  |  |  |  |  |  |  |  |  |  |
| *Mascarin (2017)*  *Brazil* | Handball  (AM) | 8 | ♂ | | 15.3 ± 0.9 | | 7 | | | ♂ | | 15.9 ± 1.2 | | *Strength Training Program*  [6-wk./3 x wk./ 30min] | | Followed their normal training routine. |
| *Niederbracht (2013)*  *USA* | Tennis  (AM) | 6 | | ♂ | | 20 ± 1.8 | | 6 | ♂ | | 18.8 ± 0.8 | | *Strength Training Program*  [5-wk./4 x wk.] | | Followed their normal preseason tennis routine. | |
|  |  |  |  | |  | |  | | |  | |  | |  | |  |
| *Romero Franco* *(2012)*  Spain | Track sprinting  (AM) | 16 | ♀ | | 22.5 ± 5.12 | | 17 | | | ♀ | | 21.18 ± 4.47 | | *Proprioceptive Training Program*  [6-wk. /3 x wk./30min.] | | Followed their normal training routine. |
| *Saraswat (2015)*  *India* | Basketball  (AM) | 15 | ♀ | | 16.3 ± 0.6 | | 15 | | | ♀ | | 16.6 ± 0.6 | | *Dynamic Balance Program*  [4-wk.] | | Conventional exercises |

Level**: **P**, professional/elite/highest level; **AM**, amateur

**BB**, basketball; **CCT**, controlled trail; **d**, days; **min.**, minutes; **mo.**, months; **N/D**, not described**;** **reps**, repetitions; **S**, season; **SE**, session(s); **VB**, volleyball; **wk.**, weeks; **yrs.**, years.

Values presented as mean ± standard deviation if not otherwise stated.
