# Supplementary material for: General versus sports-specific injury prevention programs in athletes: A systematic review on the effects on performance
Source: PLoS One. 2019 Aug 29;14(8):e0221346. doi: 10.1371/journal.pone.0221346 (PMC6715272; doi:10.1371/journal.pone.0221346)
Supplement: S2 Table — ACL, anterior cruciate ligament; BB, basketball; CPL, compliance; EX, exercise(s); KLIP Program, Knee Ligament Injury Prevention Program; LE, lower extremity; min., minutes; N/D, not described; OSTRC, Oslo Sports Trauma Research Center; PEP Program, Prevent injury and Enhance Performance Program; Pt., part/phase; reps, repetitions; s, seconds; SE, session(s); UE, upper extremity; VB, volleyball; wk., week(s); yrs., years. (DOCX) [file pone.0221346.s002.docx]

**Table S2.** *Exercise interventions applied by included studies (alphabetical order)*

| **Program**  **(Year)** | **Intervention Details** | **Dynamic warm-up** | **Plyometrics** | | **Balance** | |
| --- | --- | --- | --- | --- | --- | --- |
|  |  | **Strength** | **Agility** | | **Flexibility** | |
| ***General*** | | | | | | |
| *Chelly*  *(2010)* | 30 min. progressive plyometric training program (wk. 1 - 4: hurdle jumps; wk. 5 - 8 drop jumps). 2 x wk.  CPL: N/D | Warm-up (N/D) | | wk. 1 - 4: 1 EX (LE) wk. 5 - 8: 1 EX (LE) | | N/A |
|  |  | N/A | | N/A | | N/A |
| *Filipa*  *(2010)* | 8 Week Neuromuscular Program  (2 x wk. during in season; consisted whole body strengthening exercises with each day differing in drills.) CPL: N/D | N/A | | N/A | | N/A |
|  |  | 11 EX (LE)  4 EX (UE)  1 EX (core) | | N/A | | N/A |
| Hermassi  (2017) | 10-wk. Strength Training Program  (2 x wk.; half squat EX (80-95% 1RM; 1-3 reps and 3-6 sets) CPL: N/D | 6 EX | N/A | | N/A | |
|  |  | 1 EX (LE) | N/A | | N/A | |
| *Hoshikawa*  *(2013)* | 6-mo. Stabilization Training Program (4x wk.)  CPL: 100% | N/A | N/A | | N/A | |
|  |  | 2 EX (core)  1 EX (LE)  1 EX (UE) | 30-m sprint | | N/A | |
| *Kang*  *(2013)* | 8-wk. Balance Program using various stability ball strength and balance exercises.  CPL: N/D | N/A | | N/A | | 10 EX w/ Stab. Ball |
|  |  | N/A | | N/A | | 4 EX (UE)  3 EX (LE)  3 EX (core) |
| *Lindblom*  *(2012)* | 15 min. progressive neuromuscular warm-up program targeting core stability, balance, landing technique and proper knee alignment.  2 x wk. for 11 wk. CPL: 60% | N/A | | N/A | | N/A |
|  |  | 6 EX (LE, core) | | N/A | | N/A |
| *Prieske*  *(2014)* | 9-wk. Core Strengthening Training Program (2-3 x wk.; 4 training phases performing a variety of stability, balance and strengthening exercises performed in addition with Thera-Band and unstable surfaces). CPL: 2.3/3 mean sessions p/w. | N/A | | N/A | | N/A |
|  |  | 8 EX (core) | | N/A | | 12 EX (core) |
| *Ramirez-Campillo*  *(2014)* | 6-wk. Explosive Strength Training (2x wk./ 30min.; drop jumps from 3 various heights)  CPL: N/D | Standard warm-up | | 3 EX (LE) | | N/A |
|  |  | N/A | | N/A | | N/A |
| *Ramos Velez*  *(2014)* | 18-wk. Strength and High Intensity Program (2x wk.)  CPL: N/D | N/A | | N/A | | N/A |
|  |  | 3 EX (UE)  3 EX (LE)  1 EX (core) | | N/A | | N/A |

*Continued*

*Continued*

| **Program**  **(Year)** | **Intervention Details** | **Dynamic warm-up** | **Plyometrics** | | **Balance** | |
| --- | --- | --- | --- | --- | --- | --- |
|  |  | **Strength** | **Agility** | | **Flexibility** | |
| ***General*** | | | | | | |
| *Zech*  *(2014)* | 20 min. progressive neuromuscular warm-up including running, agility, balance, strength and plyometric components.  2 x wk. for 10 wk.  CPL: 86% | N/A | | 3 EX (LE, core) | | 3 EX (LE, core) |
|  |  | 3 EX (LE, core) | | 6 drills | | N/A |
| *Zouita*  *(2016)* | 12-wk. Progressive Strength Training (2-3 x wk./ 90min.)  CPL: N/D | N/A | | N/A | | N/A |
|  |  | 1 EX (LE)  2 EX (UE)  1 EX (core) | | N/A | | N/A |
| ***Mixed*** | | | | | | |
| *FIFA 11+ (2013)*^#^ Steffen  *(2017)” Baeza*  *(2013)* Daneshjoo* | 20 min. three component warm-up program with 3 difficulty stages.  2 - 3 x wk. for 2 mo.^#^  3 x wk. for 6 wk.”  24 SE over 8 wk.*  CPL: 72%^#^; 100%“; N/D* | 6 running drills^#^”* | | 2 jumping EX^#^”* | | 1 EX^#^”* |
|  |  | 3 EX (LE, core)^#^”* | | 2 drills^#^”* | | N/A^#^”* |
| *F-MARC 11/The 11*  *(2008)*’’ Steffen  *(2008)^#^*  Kilding | 10 – 15 min. warm-up program with 10 evidence-based exercises focusing on core stability, balance, dynamic stabilization and eccentric hamstring strength.  3 x wk. for 10 wk.’’  5 x wk. for 6 wk.^#^ CPL: 73.3%’’; ø 23.5^#^ | Jogging’’  N/D^#^ | | 2 jumping EX’’^#^ | | 4 EX (ball, pair)^#^ 4 EX (ball, pair, balance mat)’’ |
|  |  | 2 EX (LE, core)^#^  3 EX (LE, core)’’ | | 1 drill’’^#^ | | N/A’’^#^ |
| *HarmoKnee*  *(2013) Daneshjoo* | Harmoknee Injury Prevention Program  (3 x wk./ 8 wk.; 20-25 min. per SE) CPL: N/D | 9 EX + Activation (10min.) | | N/A | | 3 EX (core) |
|  |  | 3 EX (LE) | | 4 EX (LE) | |  |
| *Ondra*  *(2017)* | 20-wk. Neuromuscular Training Program (3x wk./ 20min.)  CPL: N/D | Whole body warm-up | | 1 EX (LE) | | N/A |
|  |  | 1 EX (core)  2 EX (LE)  1 EX (UE) | | N/A | | N/A |
| *PEP Program*  *Vescovi & VanHeest*  *(2009)* | 30 min. PEP Program warm-up focuses on ACL injury prevention by stretching, strengthening, plyometrics, agilities and avoidance of high-risk positions.  Replacement exercises were given.  3 x wk. 12 wk.  CPL: 92% | 3 jogging and running drills | | 5 jump EX | | N/A |
|  |  | 3 EX (LE) | | 3 drills | | 5 EX (LE) |
| *Rubley*  *(2011)* | 14-wk. Plyometric Training (2-3 x wk.)  CPL: N/D | N/A | | 4 EX (LE) | | N/A |
|  |  | N/A | | N/A | | N/A |

*Continued*

| **Program**  **(Year)** | **Intervention Details** | **Dynamic warm-up** | **Plyometrics** | | **Balance** | |
| --- | --- | --- | --- | --- | --- | --- |
|  |  | **Strength** | **Agility** | | **Flexibility** | |
| ***Sports Specific*** | | | | | | |
| *Asadi*  *(2015)* | 6-wk. Plyometric Program (2 x wk./ 60min.; consisted of different jumps and drops from a 20 and 45cm box during in season)  CPL: N/D | N/A | 3 EX (LE) | | N/A | |
|  |  | N/A | N/A | | N/A | |
| *Fachina*  *(2017)* | 8-wk. Plyometric Training (3x wk.)  CPL: N/D | N/A | 3 EX (LE) | | N/A | |
|  |  | 5 EX (LE) | N/A | | N/A | |
| *Hermassi*  *(2015)* | 8-wk. In Season Resistance Training (3 x wk.)  CPL: N/D |  | |  | |  |
|  |  | 3 EX (UE) | |  | |  |
| *Jakeman*  *(2016)* | 4-wk. High Intensity Training (2 x wk.)  CPL: N/D | N/A | N/A | | N/A | |
|  |  | Progressive hill sprints (8% incline) | N/A | | N/A | |
| *Julien*  *(2008)* | 3-wk. Squat or Coordination Training  CPL: N/D | N/A | | N/A | | N/A |
|  |  | N/A | | Agility and coordination course | | N/A |
| *Mascarin*  *(2017)* | 6-week Strength Training Program with Thera-Band  3 x wk./ 30 min.; 3 sets x 10 reps, 30secs rest; resistance was progressively increased)  CPL: 100% | 2 EX (UE) | N/A | | N/A | |
|  |  | N/A | N/A | | N/A | |
| *Romero Franco*  *(2012)* | 30 min. progressive sprinter specific proprioception training program consisting of 2 phases (3 weeks each) using a range of lower limb movements with the BOSU and Swiss ball.  3 x wk. for 6 wk. CPL: N/D | N/A | | N/A | | wk. 1 - 3: 5 EX (LE) wk. 4 - 6: 5 EX (3 DB, 2 ankle weight) |
|  |  | N/A | | N/A | | N/A |
| *Saraswat*  *(2015)* | 4-wk. Dynamic Balance Program  CPL: N/D | 1 EX (LE) | 1 EX (LE; foam pads, eyes open, medicine ball throws, dynamic movements) | | N/A | |
|  |  | N/A | N/A | | N/A | |
| *Niederbracht*  *(2008)* | 5-wk. Strength Training Program (4 x wk.; 3 sets x 15 reps)  CPL: N/D | N/A | N/A | | N/A | |
|  |  | 5 EX (UE) | N/A | | N/A | |

**ACL**, anterior cruciate ligament; **BB**, basketball; **CPL**, compliance; **EX**, exercise(s); **KLIP** **Program**, Knee Ligament Injury Prevention Program; **LE**, lower extremity; **min.**, minutes; **N/D**, not described; **OSTRC**, Oslo Sports Trauma Research Center; **PEP Program**, Prevent injury and Enhance Performance Program; **Pt.**, part/phase; **reps**, repetitions; **s**, seconds; **SE**, session(s); **UE**, upper extremity; **VB**, volleyball; **wk.**, week(s); **yrs.**, years
