# Supplementary material for: General versus sports-specific injury prevention programs in athletes: A systematic review on the effects on performance
Source: PLoS One. 2019 Aug 29;14(8):e0221346. doi: 10.1371/journal.pone.0221346 (PMC6715272; doi:10.1371/journal.pone.0221346)
Supplement: S3 Table — “Statistically significant difference within groups (pre- post-test). * Statistically significant difference between intervention and control group (pre- post-test). 5JT, 5 jump test; BAL, balance training group; BESS, Balance error scoring system; CG control, general gravity center control; CMJ, counter movement jump; COP, center of pressure; DEO, distance covered by the center of pressure with eyes open; DJ, drop jump; ECC, Eccentric strengthening exercises; ER, external rotation; IR, internal rotation; MAS, maximal aerobic speed; MSFT, multi-stage fitness test; PLYO, plyometric training group; RAST, running-based anaerobic sprint test; RJ; rebound jump,; RombergD, Romberg index about distance; RombergS, Romberg index about surface; RombergSp, Romberg index about speed; SE, session(s); SEBT, Star Excursion Balance Test; SEO, surface covered by the center of pressure with eyes open; SLH, single leg hop; SLTH, single leg triple crossover hop; SLS, single leg squat; SpEO, speed of center of pressure movement with eyes open; THDT, Triple hop distance; TTS, time to stabilization; UNS, unstable surface exercises; VDJ, vertical drop jump; VO2max, maximal oxygen uptake; XEO, mean position center of pressure in the medial-lateral plane with eyes open; YEO, mean position center of pressure in the anterior-posterior plane with eyes open; YYIRTL1, Yo-Yo intermittent recovery test level 1; Values presented as mean ± standard deviation or Δ% or if not otherwise stated. (DOCX) [file pone.0221346.s003.docx]

**Table S3.** *Results and conclusion of included studies (alphabetical order)*

| **Study** | **Outcome measures** | **Results** | | | | **Conclusion** |
| --- | --- | --- | --- | --- | --- | --- |
|  |  | **Intervention** | | **Control** | |  |
|  |  | **Pre** | **Post** | **Pre** | **Post** |  |
| ***General*** | | | | | | |
| *Chelly*  *(2010)* | **Absolute Power [W] Maximal Force [N]** **Squat jump**  *Height [m]  Velocity [mm.s^-1^]*  *Force [N]  Power [W]* **CMJ jump**  *Height [m]  Velocity [mm.s^-1^]*  *Force [N]  Power [W]*  **Sprint velocities *[mm.s^-1^]*** | 711 ± 84 84.4 ± 8.5  0.36 ± 0.03 2.4 ± 0.1 1540 ± 141 1398 ± 145  0.40 ± 0.03 2.5 ± 0.1 1347 ± 111 1673 ± 147 8.2 ± 0.2 | 743 ± 87’’* 83.0 ± 6.1’’  0.39 ± 0.03’*’ 2.6 ± 0.1’’* 1590 ± 168 1511 ± 197  0.41 ± 0.03’*’ 2.6 ± 0.1’’* 1372 ± 108 1753 ± 170’’* 9.0 ± 0.2’’* | 680 ± 128 84.6 ± 4.4  0.37 ± 0.02 2.1 ± 0.2 1504 ± 167 1451 ± 150  0.39 ± 0.02 2.2 ± 0.2 1313 ± 133 1612 ± 176 7.8 ± 0.3 | 682 ± 129 84.5 ± 3.7  0.37 ± 0.02 2.1 ± 0.2 1589 ± 160 1464 ± 161  0.39 ± 0.02 2.2 ± 0.1 1345 ± 147 1612 ± 161 8.0 ± 0.3 | Plyometric training (2 x wk.) improves important performance components and should be implemented as part of soccer training. |
| *Filipa*  *(2010)* | **SEBT**  *Anterior (right)*  *Anterior (left)*  *Posteromedial (right)*  *Posteromedial (left)*  *Posterolateral (right)*  *Posterolateral (left)* | 68.2 ± 11.0  70.4 ± 6.6  101.4 ± 16.4  98.1 ± 15.6  87.6 ± 13.6  89.8 ± 13.0 | 71.7 ± 5.8  70.4 ± 6.1  107.4 ± 9.6  105.6 ± 12.9*  99.2 ± 10.0*  99.2 ± 7.3* | 62.9 ± 4.4  63.7 ± 6.8  96.9 ± 8.3  102.2 ± 11.7  94.1 ± 10.2  92.4 ± 7.5 | 61.7 ± 4.4  59.7 ± 6.5  95.0 ± 8.8  97.1 ± 9.2  93.7 ± 7.9  91.4 ± 4.9 | Female soccer players demonstrated an improved performance on the SEBT after NMTP that focused on core stability and lower extremity strength. |
| *Hermassi*  *(2017)* | **Maximal Lower Limb load [kg]**  **CMJ [cm]**  **SJ [cm]**  **YYIRTL [km.h^-1^]**  **RSA Best Time [s]**  **RSA Mean Time [s]**  **RSA Total Time [s]** | 206.0 ± 12.50  43.8 ± 0.75  41.8 ± 1.19  17.0 ± 0.69  6.74 ± 0.31  6.93 ± 0.31  40.5 ± 3.39 | 236.0 ± 9.50”*  47.0 ± 0.95”*  44.3 ± 0.75”*  18.2 ± 0.49”*  6.2 ± 0.12”*  6.5 ± 0.08”  39.1 ± 0.45 | No significant difference between pre/post | | Additional strength training enhances maximal lower limb strength and jumping and repeated sprint ability. |
| *Hoshikawa*  *(2013)* | **HET [Nm]**  **HFT [Nm]**  **SJ [cm]**  **CMJ [cm]**  **15m Sprint [s]** | 112.5 ± 34.0  96.3 ± 26.7  33.3 ± 3.9  45.0 ± 4.5  2,6 ± 0,1 | 155.3 ± 44.4”*  108.2 ± 26.2”  34.6 ± 2.6”  47.8 ± 3.5”  2,5 ± 0,1” | 128.1 ± 28.6  95.1 ± 16.4  32.5 ± 3.5  44.4 ± 4.2  2,6 ± 0,1 | 147.5 ± 25.9”  107.2 ± 15.5”  32.7 ± 3.9  44.9 ± 5.7  2,607 ± 0.114” | The extra training did not effect sprint time; however, SJ and CMJs were improved in addition to hip extension torque. |

*Continued*

*Continued*

| **Study** | **Outcome measures** | **Results** | | | | **Conclusion** |
| --- | --- | --- | --- | --- | --- | --- |
|  |  | **Intervention** | | **Control** | |  |
|  |  | **Pre** | **Post** | **Pre** | **Post** |  |
| ***General*** | | | | | | |
| *Kang*  *(2013)* | **Sit-up [n/30s]**  **Push-up [n/30s]**  **Grip Strength [kg]**  **Vertical Jump [cm]**  **Side Step [n/30s]**  **Reaction Time [s]**  **One-legged Stand (eyes close) [s]**  **Sit and Reach [cm]**  **Back Hyperextension [cm]** | HS: 23.50 ± 0.77  MS: 28.11 ± 0.94  HS: 39.62 ± 2.39  MS: 36.55 ± 2.68”  HS: 54.79 ± 2.55  MS: 38.81 ± 3.08  HS: 53.5 ± 2.88  MS: 48.55 ± 2.31  HS: 30.37 ± 2.56  MS: 34.22 ± 1.47  HS: 0.21 ± 0.01  MS: 0.25 ± 0.01  HS: 22.12 ± 3.4*  MS: 38.44 ± 7.63  HS: 14.57 ± 1.63  MS: 16.55 ± 1.12  HS: 58.81 ± 2.76*  MS: 58.38 ± 2.16 | HS: 25.00 ± 1.09  MS: 27.55 ± 1.55  HS: 38.25 ± 2.92  MS: 36.89 ± 2.26”  HS: 54.81 ± 2.31  MS: 40.35 ± 2.75  HS: 51.87 ± 2.83  MS: 49.33 ± 2.47  HS: 35.12 ± 1.18  MS: 31.89 ± 1.23  HS: 0.24 ± 0.01  MS: 0.22 ± 0.01  HS: 39.75 ± 6.78  MS: 48 ± 10.93  HS: 15.88 ± 1.74  MS: 18.82 ± 1.31  HS: 66.90 ± 2.33  MS: 64.02 ± 1.51 | 27.6 ± 1.08  26 ± 0.5  30.4 ± 3.41  17 ± 3.55  51.26 ± 2.47  39.82 ± 4.41  52.3 ± 2.42  41.6 ± 2.46  36.1 ± 1.39  35 ± 1.25  0.209 ± 0.01  0.233 ± 0.02  14.1 ± 3.19*  22.2 ± 5.11  20.37 ± 1.61  19.44 ± 2.92  60.36 ± 1.16  58.54 ± 1.82 | | The results of this 8-week program suggest that balance and flexibility are improved and therefore may enhance athletes sport performance. |
| *Lindblom*  *(2012)* | **SEBT [cm]**  *Composite score right  Composite score left* **CMJ [cm] THDT [cm] Illinois agility test [s] 10-m sprint [s] 20-m sprint [s]** | 109.0 ± 8.0  109.8 ± 7.9 25.5 ± 3.7  523 ± 55.1  17.43 ± 0.89 2.02 ± 0.11 3.58 ± 0.19 | 108.9 ± 7.4  108.4 ± 6.6 25.0 ± 4.0 506 ± 52.5’’  17.50 ± 0.83  2.02 ± 0.12 3.57 ± 0.23 | 105.5 ± 7.3  106.2 ± 6.9 24.5 ± 3.1 527 ± 53.3  17.39 ± 0.71 2.00 ± 0.08 3.52 ± 0.15 | 108.0 ± 7.8 109.1 ± 8.2’’ 24.6 ± 3.2 525 ± 43.0  17.20 ± 0.83’’ 2.01 ± 0.10 3.52 ± 0.17 | The warm-up did not improve any indices of performance. |
| *Prieske*  *(2015)* | **Trunk Muscular Strength**  *Flexor MIF (N)*  *Extensor MIF (N)*  *Flexor MAV [%]*  *Extensor MAV [%]*  **Athletic Performance**  *CMJ [cm]*  *0-10m Time [s]*  *10-20m Time [s]*  *0-20m Time [s]*  *T-test Time [s]*  *Kicking Performance [km.h^-1^]* | **Unstable surface**  624.40 ± 99.6  591.40 ± 67.10  53.20 ± 11.40  63.20 ± 3.20  34.00 ± 3.40  1.71 ± 0.06  1.28 ± 0.05  2.99 ± 0.11  9.70 ± 0.30  101.30 ± 6.80 | 617.70 ± 97.60  614.00 ± 115.10  55.60 ± 12.70  62.40 ± 6.00  34.30 ± 2.70  1.73 ± 0.06  1.25 ± 0.02  2.97 ± 0.07  9.70 ± 0.40  103.40 ± 6.30 | **Stable Surface**  656.50 ± 92.30  603.10 ± 98.80  47.90 ± 9.50  60.50 ± 4.90  36.00 ± 3.40  1.69 ± 0.04  1.27 ± 0.02  2.96 ± 0.05  9.70 ± 0.40  107.90 ± 5.80 | 681.00 ± 89.30  644.00 ± 92.60  56.80 ± 4.40  62.30 ± 5.40  35.50 ± 3.20  1.72 ± 0.08  1.22 ± 0.04  2.95 ± 0.11  9.70 ± 0.50  107.50 ± 6.10 | Trunk muscle strength, sprint and kicking performance improved following both training protocols when conducted in combination with regular soccer training. |

*Continued*

*Continued*

| **Study** | **Outcome measures** | **Results** | | | | **Conclusion** |
| --- | --- | --- | --- | --- | --- | --- |
|  |  | **Intervention** | | **Control** | |  |
|  |  | **Pre** | **Post** | **Pre** | **Post** |  |
| ***General*** | | | | | | |
| *Ramirez Campillo*  *(2014)* | **20m [s]**  **2.4km [min]**  **CMJA [cm]**  **DJ 20cm [cm/m.s^-1^]**  **DJ 40cm [cm/m.s^-1^]** | 3.92 ± 0.3  7.6 ± 0.7  36.1 ± 5.6  0.157 ± 0.049  0.156 ± 0.041 | 3.83 ± 0.3”*  7.3 ± 0.8”*  39.3 ± 7”  0.177 ± 0.044”  0.182 ± 0.051”* | No significant difference between pre/post | | Explosive strength and endurance training could be advantageous for middle and long-distance runners, especially characterized by sprint actions. |
| *Ramos Velez*  *(2014)* | **CMJ [cm]**  **Bench Press [kg]**  **Squat [kg]**  **Throwing Velocity [km.h^-1^]**  **Swim Sprint 20m [s]** | 34.48 ± 1.47  86.06 ± 13.6  77.81 ± 16.3  64.00 ± 6.7  11.51 ± 0.6 | 36.86 ± 4.9”  95.12 ± 15.5”*  88.87 ± 16.5”*  65.76 ± 7.3”  11.25 ± 0.6”* | No significant difference between pre/post | | Strength training had a positive effect on qualities highly related to waterpolo. |
| *Zech*  *(2010)* | **SEBT- ∆ [%]**  *Anterior  Postero-lateral  Postero-medial* **TTS- ∆ [%]**  *Medial-Lateral*  *Anterior-Posterior* **BESS- ∆ (total; [%])** **COP sway- ∆ (total; [%])** | −3.3 ± 4.8 1.6 ± 5.9 0.2 ± 5.4  -15.3 ± -15.7  -5.2 ± 18.4 -27.5 ± -27.1 -0.9 ± 13.3 | 3.3 ± 12.9 6.7 ± 8.2 3.8 ± 5.1  -15.2 ± -15.8 -3.7 ± 22.8  -69.3 ± -10.3* -11.1 ± 9.5 | −0.9 ± 5.5 4.6 ± 6.7  1.4 ± 7.4  -7.2 ± 30.1 -0.6 ± 32.7 -26.5 ± 23.2  2.6 ± 15.4 | 3.6 ± 7.5  6.1 ± 11.0 4.8 ± 9.4  -10.7 ± 37.4 -1.0 ± 23.1 -31.8 ± 22.1 -10.3 ± 10.2 | Improvement of some indicators of postural control. |
| *Zouita*  *(2016)* | **Sprint time**  *10m [s]*  *20m [s]*  *30m [s]*  **5JT [m]**  **SJ [m]**  **CMJ [m]**  **DJ [m]**  **T-test [s]**  **YYIRTL1 [m]**  **MAS [km.h^-1^]** | 2.1 ± 0.12  3.3 ± 1.0  4.9 ± 0.3  10.2 ± 0.5  27.8 ± 6.5  34.3 ± 3.8  29.1 ± 5.3  9.0 ± 0.6  1,015,4 ± 372  14.2 ± 0.9 | 2.0 ± 0.1*  3.0 ± 0.5”*  4.8 ± 0.3  11.3 ± 0.6”  35.1 ± 5.2”*  41.9 ± 3.1”  29.3 ± 3.9  7.2 ± 0.3”  1,907.7 ± 443.7”*  16.5 ± 1.1”* | 2.2 ± 0.2  3.7 ± 0.2  5.0 ± 1.1  10.5 ± 0.6  29.2 ± 3.3  33.3 ± 3.6  26.7 ± 3.6  8.8 ± 0.6  993.8 ± 347.7  14.1 ± 0.9 | 2.1 ± 0.2”  3.3 ± 0.4”  4.7 ± 0.3  11.2 ± 0.8  31.2 ± 4.4  40.4 ± 2.9”  30.4 ± 3.6”  7.4 ± 0.3”  1,576.9 ± 320.6”  15.7 ± 0.8” | The program had many positive outcomes compared to CG in many football performance |

*Continued*

*Continued*

| **Study** | **Outcome measures** | **Results** | | | | **Conclusion** |
| --- | --- | --- | --- | --- | --- | --- |
|  |  | **Intervention** | | **Control** | |  |
|  |  | **Pre** | **Post** | **Pre** | **Post** |  |
| ***Mixed*** | | | | | | |
| *Baeza*  *(2017)* | **Functional Movement Screen (0-3)**  *Squatting*  *Stepping*  *Lunging*  *Reaching*  *Leg Raising*  *Push-up [∆]*  *Rotatory Stability*  *Total* | 1.5 ± 0.5  1.6 ± 0.7  1.4 ± 0.5  2.6 ± 0.7  2.0 ± 0.4  1.9 ± 0.8  2  13.1 ± 1.3 | 1.8 ± 0.4  1.5 ± 0.5  2 ± 0.4”  3  2  2.2 ± 0.9  2  14.5 ± 1.3 | 1.6 ± 0.5  1.4 ± 0.5  1.8 ± 2.1  2.8 ± 0.4  1.9 ± 0.3  1.5 ± 0.8  2  13.1 ± 1.8 | 1.2 ± 0.4  1.5 ± 0.7  2.1 ± 0.7  2.8 ± 0.4  1.8 ± 0.4  1.5 ± 0.7  2  12.9 ± 2.3 | FIFA 11+ was not an effective way to improve fundamental movement patterns amongst young male footballers. |
| *Daneshjoo*  *(2013)* | **Concentric Peak Torque (Leg)**  *Dom Q_con_ 60°.s^-1^ [N.m]*  *Non- Dom Q_con_ 60°.s^-1^ [N.m]*  *Dom Q_con_ 180°.s^-1^ [N.m]*  *Non- Dom Q_con_ 180°.s^-1^ [N.m] Dom Q_con_ 300°.s^-1^ [N.m]*  *Non- Dom Q_con_ 300°.s^-1^ [N.m]*  *Dom H_con_ 60°.s^-1^ [N.m]*  *Non- Dom H_con_ 60°.s^-1^ [N.m]*  *Dom H_con_ 180°.s^-1^ [N.m]*  *Non- Dom H_con_ 180°.s^-1^ [N.m] Dom H_con_ 300°.s^-1^ [N.m]*  *Non- Dom H_con_ 300°.s^-1^ [N.m]*  **Eccentric Peak Torque (Leg)**  *Dom H_con_ 120°.s^-1^ [N.m]*  *Non- Dom H_con_ 120°.s^-1^ [N.m]*  *Dom Q_con_ 120°.s^-1^ [N.m]*  *Non- Dom Q_con_ 120°.s^-1^ [N.m]* | **FIFA 11 [∆%]**  22.6  6.0  20.6  17.3  27.7  22.0  22.0  22.3  21.4  15.7  22.1  15.2  -7.5  -10.1  7.2  13.1 | **HarmoKnee [∆%]**  36.6  31.3”  36.2  31.7”  28.0  20.1  32.5  21.1  31.3  19.3  14.3  10.3  5.1  7.1  -0.1  0.7 | **Control ∆%**  5.2  -13.9  2.8  1.3  4.0  -3.5  6.3  -6.4  2.1  7.9  -3.9  3.9  -1.1  -6.9  4.8  -7.8 | | The HarmoKnee is useful for concentric quadriceps strength. FIFA11 and HarmoKnee are useful for improvements in hamstring strength. |
| *Kilding*  *(2008)* | **3 step jump [m]**  **CMJ [m]**  **Agility [s]**  **Core Stability [s]**  **20m sprint [s]** | 4.61 ± 0.34  0.33 ± 0.13  14.81 ± 0.72  45.2 ± 16.1  3.60 ± 0.35 | 4.77 ± 0.20*****  0.35 ± 0.15*****  14.52 ± 0.53  64.5 ± 10.2  3.52 ± 0.36***** | 4.80 ± 0.23 0.36 ± 0.11 15.51 ± 0.93 34.30 ± 14.90 3.74 ± 0.07 | 4.79 ± 0.23 0.36 ± 0.05 15.43 ± 0.86 43.30 ± 11.90 3.81 ± 0.98 | Program should be considered for young players for both physical development and possible injury prevention. |

*Continued*

*Continued*

| **Study** | **Outcome measures** | **Results** | | | | **Conclusion** |
| --- | --- | --- | --- | --- | --- | --- |
|  |  | **Intervention** | | **Control** | |  |
|  |  | **Pre** | **Post** | **Pre** | **Post** |  |
| ***Mixed*** | | | | | | |
| *Ondra*  *(2017)* | **Mean COP** **Medial Lateral**  *Dom Limb [cm.s^-1^]*  *Non- Dom Limb [cm.s^-1^]*  **Mean COP** **Anterior-Posterior**  *Dom Limb [cm.s^-1^]*  *Non- Dom Limb [cm.s^-1^]*  **Mean COP Total Velocity**  *Dom Limb [cm.s^-1^]*  *Non- Dom Limb [cm.s^-1^]* | 3.57 ± 0.90  3.54 ± 0.72  3.51 ± 0.85  3.32 ± 0.70  5.45 ± 1.27  5.24 ± 1.00 | 2.85 ± 0.45*  2.67 ± 0.30*  2.81 ± 0.34  2.70 ± 0.34*  4.43 ± 0.57*  4.20 ± 0.50* | 3.36 ± 0.45  3.29 ± 0.59  3.21 ± 0.38  2.99 ± 0.54  5.15 ± 0.53  4.93 ± 0.79 | 3.68 ± 0.55  3.84 ± 0.64  3.35 ± 0.68  3.48 ± 0.68  5.54 ± 0.95  5.75 ± 0.99 | Proprioceptive and neuromuscular training had a positive effect of stability in both limbs in basketball players. |
| *Rubley*  *(2011)* | **Average Kicking Distance [m]**  **Average VJ Height [cm]** | 25.9 ± 2.6  39.6 ± 8.2 | 33 ± 3.7”*  47 ± 8.1”* | 27.6 ± 2.5  39.4 ± 8.3 | 23.3 ± 3.7  36.8 ± 6.2 | Plyometric training is proven to be extremely effective in improving indicators of explosive power in adolescent athletes. |
| *Steffen*  *(2008)* | **∆ Isokinetic leg *[Nm]***  *Q con 60° s^-1^  Q ecc 60° s^-1^   Q con 240° s^-1^   H con 60° s^-1^   H ecc 60° s^-1^   H con 240° s^-1^*  **∆** **Isometric leg *[Nm]***  *Q iso 30°*  *Q iso 60°   Q iso 90°   H iso 30°   H iso 60°   H iso 90°* | 3.1 ± 11.8  4.1 ± 20.1 -0.2 ± 10.7 -0.9 ± 8.6 -1.4 ± 8.6 1.1 ± 6.9  1.9 ± 10.5 4.1 ± 16.1 4.1 ± 16.7 3.8 ± 15.2 4.7 ± 11.8 -0.5 ± 11.8 | | 1.6 ± 10.3 14.1 ± 26.3 0.9 ± 10.6 -2.1 ± 5.4 -4.1 ± 12.0 -1.0 ± 9.0  0.2 ± 7.7 8.3 ± 16.8 16.4 ± 18.6 -1.5 ± 12.9 4.9 ± 12.6 0.6 ± 10.1 | | No effects were observed between groups.  Warm-up program is not necessarily related to performance enhancement. |

*Continued*

*Continued*

| **Study** | **Outcome measures** | **Results** | | | | **Conclusion** |
| --- | --- | --- | --- | --- | --- | --- |
|  |  | **Intervention** | | **Control** | |  |
|  |  | **Pre** | **Post** | **Pre** | **Post** |  |
| ***Mixed*** | | | | | | |
| *Steffen*  *(2008)* | **∆** **Isometric ab- & adduction [kg]**  *Kicking foot ext.   Kicking foot flex.*  *Stand foot*  **∆ Jumping ability [cm]**  *CMJ   VDJ*  *15-s RJ*  **∆ 40-m sprint [s] ∆ Shuttle run w/ball ∆ Long distance kick [m]** | 0.6 ± 1.7 0.4 ± 3.1 -1.8 ± 2.4  -0.7 ± 1.4 -0.3 ± 1.9 -0.8 ± 2.0 -0.04 ± 0.11*  -0.68 ± 0.81  -1.1 ± 3.4 | | 0.9 ± 2.2 2.1 ± 2.9 -2.0 ± 1.8  -1.3 ± 1.1 -0.7 ± 2.1 -0.5 ± 2.6 0.01 ± 0.11  -0.71 ± 0.69  -0.9 ± 2.3 | |  |
| *Steffen*  *(2013)* | **Single leg balance [s]**  *Left  Right* **SEBT [cm]**  *Anterior left  Postero-lateral left  Postero-medial left*  *Anterior right  Postero-lateral right  Postero-medial right* **SLTH left [cm] SLTH right [cm] Bar jumps [reps]** | Mean  5.51 ± 4.49 5.68 ± 3.31  75.4 ± 6.3 81.8 ± 7.9 79 ± 9.5 74.9 ± 6.7 80.7 ± 8.1 79.4 ± 8.7 445.9 ± 67.4 453.1 ± 68.4 38.9 ± 3.7 | ∆ 0.46’’ 0.23’’  6.9’’* 6.1’’ 5.8’’ 7.6’’* 6.0’’ 5.7’’  11.1’’  15.6’’ -3.1* | Mean 6.52 ± 6.49 5.60 ± 3.15  76.4 ± 6.6 80.8 ± 7.2 78.5 ± 8.3 75.6 ± 6.5 79.6 ± 7.3 78.5 ± 8.5 431.3 ± 56.9 443.6 ± 61.2 34.7 ± 5.4 | ∆ −1.37 −0.09   1.9 4.6 3.4 3.2 4.5  4.4 4.1  3.4  0.5 | High adherence to FIFA 11+ and significant individual improvements in functional balance. |
| *Vescovi & VanHeest*  *(2010)* | **Sprint times [s]**  *9.1-m  18.2-m  27.3-m  36.6-m* **CMJ [cm] Illinois agility test [s] Pro-Agility [s]** | No difference  No difference  No difference  No difference | | Slower*  Slower*  Lower* | | Minor significant sprint improvements.  PEP program demonstrated no effect on CMJ or agility. |

*Continued*

*Continued*

| **Study** | **Outcome measures** | **Results** | | | | **Conclusion** |
| --- | --- | --- | --- | --- | --- | --- |
|  |  | **Intervention** | | **Control** | |  |
|  |  | **Pre** | **Post** | **Pre** | **Post** |  |
| ***Sports Specific*** | | | | | | |
| *Asadi*  *(2015)* | **SEBT performance [cm]**  *Anterior*  *Anteromedial*  *Anterolateral*  *Medial*  *Lateral*  *Posterior*  *Posteromedial*  *Posterolateral* | 98.19 ± 5.05  83.29 ± 8.07  99.68 ± 7.15  71.41 ± 7.48  93.3 ± 7.92  87.6 ± 11.62  80.81 ± 12.29  91.64 ± 10.41 | 103.02 ± 3.63”*  88.32 ± 7.19”*  104.04 ± 6.38”*  74.12 ± 6.67”*  97.08 ± 5.85”*  90.82 ± 10.64”*  83.93 ± 10.73”  94.17 ± 10.61” | No significant difference between pre/post | | Plyometrics is recommended to enhance athlete’s postural control. |
| *Fachina (2017)* | **Modified RAST**  *Mean Power [W]*  *Maximum Power [W]*  *Fatigue Index [%]*  *Total Time [s]*  **Modified t-test**  *Best Time [s]* | 30.0 ± 8.7  37.1 ± 12.4  9.2 ± 5.4  89.5 ± 8.8  11.0 ± 1.2 | 100.7 ± 30.3”*  115.4 ± 32.8”*  6.2 ± 2.6  59.1 ± 5.1”*  9.0 ± 0.2”* | 30.1 ± 12  36.3 ± 16.4  7.9 ± 5.3  89.7 ± 8.8  11.8 ± 1.2 | 47.2 ± 30.4”  54.6 ± 35.7”  6.0 ± 2.1  80.7 ± 14.8”  11.0 ± 0.9” | Implementing plyometric training has shown to vastly improve anaerobic sprinting activity and agility. |
| *Hermassi*  *(2015)* | **Standing Throw [m.s^-1^]**  **Jump Throw [m.s^-1^]**  **Throw With Run [m.s^-1^]**  **Pull Over [kg]**  **Bench Press [kg]** | Reg: 19.8 ± 1.7  M.Ball: 19.3 ± 1  Reg: 23.1 ± 1.3  M.Ball: 23.1 ± 1.2  Reg: 24.9 ± 1.4 M.Ball: 25.6 ± 1.1  Reg: N/D  M.Ball: N/D  Reg: N/D  M.Ball: N/D | Reg: N/D  M.Ball: +24.2%”*  Reg: +16.7%”*  M.Ball: +22.1%”*  Reg: N/D  M.Ball: +22.4%””  Reg: +20.3%”*  M.Ball: +20.3%”*  Reg: +13%”*  M.Ball: +19.1%”* | No significant changes for any performance measure. | | Male handball players can improve 3 types of ball throwing velocities, maximal upper body strength and power by implementing a medicine ball throwing program. |
| *Jakeman*  *(2016)* | **Total Sprint Time [s]**  **Fastest Sprint Time [s]**  **Slowest Sprint Time [s]**  **Mean Sprint Time [s]**  **Shuttle Sprint (w/stick)**  **Shuttle Sprint (w/stick & ball)**  **Slalom (w/stick)**  **Slalom (w/stick & ball)** | 27.3 ± 1.0  4.21 ± 0.3  4.8 ± 0.2  4.5 ± 0.2 | 23.1 ± 1.4”*  3.7 ± 0.4”*  4.1 ± 0.3”*  3.9 ± 0.2”*  + 2.8% (post)*  + 0.7% (post)*  No difference  No difference | No significant difference between pre/post | | A short period of high intensity training can significantly improve hockey related performance measures. |

*Continued*

*Continued*

| **Study** | **Outcome measures** | **Results** | | | | **Conclusion** |
| --- | --- | --- | --- | --- | --- | --- |
|  |  | **Intervention** | | **Control** | |  |
|  |  | **Pre** | **Post** | **Pre** | **Post** |  |
| ***Sports Specific*** | | | | | | |
|  |  |  |  |  |  |  |
| *Julien*  *(2008)* | **7.32m Sprint [s]**  **10m Sprint [s]**  **Shuttle Test [s]**  **Timed Circuit Test [s]** | Time interaction but no group interaction.  No intergroup effect.  Significant effects for groups and test session but not compared to each other. |  |  |  | It appears that soccer-specific training composed of exercise circuits specifically adapted to the different types of effort actually used in match play can enhance agility and co-ordination. |
| *Mascarin*  *(2017)* | **Concentric IR (Arm)**  *Dom PT 60°/s [N.m]*  *Non-Dom PT 60°/s [N.m]*  **Concentric ER (Arm)**  *Dom PT 60°/s [N.m]*  *Non-Dom PT 60°/s [N.m]*  *Dom PT 240°/s [N.m]*  *Non-Dom PT 240°/s [N.m]*  **Eccentric ER (Arm)**  *Dom PT 240°/s [N.m]*  *Non-Dom PT 240°/s [N.m]*  **Ball throwing velocity (Arm)**  *Dom [km****.****h^-1^]*  *Non-Dom [km****.****h^-1^]* | 29.4 ± 1  29.4 ± 1  18 ± 0.8  18.08 ± 0.8  16.4 ± 1.2  18.5 ± 0.8  30.8 ± 1.2  27.6 ± 3.5  49.0 ± 2.4  38.1 ± 2.5 | 30.6 ± 1.8  29.1 ± 1.3“  21.3 ± 1.0”  21.1 ± 1.3*”  15.4 ± 1.1  17.5 ± 1.9  30.2 ± 1.8  36.0 ± 1.9*”  52.5 ± 2.2”  37.2 ± 1.1 | 32 ± 1.3  26.8 ± 1.0  21.2 ± 1.4  15.6 ± 0.7  17.8 ± 1.7  18.6 ± 1.9  31.5 ± 1.7  29.9 ± 3.3  53.3 ± 1.8  36.6 ± 1.0 | 26.5 ± 1.2  23.8 ± 0.9  22.9 ± 1.8  16.1 ± 1.1  15.6 ± 2.9  17.1 ± 0.8  32.5 ± 1.8  29.2 ± 1.0  52.6 ± 1.6  40.4 ± 1.2” | The strength training improves muscular strength of external rotator muscles. |
| *Niederbracht*  *(2013)* | **Con internal total work (J)**  **Ecc external total work (J)**  **Con internal mean peak force (N)**  **Ecc external mean peak force (N)** | 89.09 ± 18.16*  96.16 ± 37.13”  50.32 ± 5.86*  59.02 ± 11.98” | 128.22 ± 65.60*  170.99 ± 72.98”*  63.23 ± 20.77  76.53 ± 19.28” | 53.54 ± 14.2*  75.43 ± 44.67  40.3 ± 8.51*  53.25 ± 30.9 | 68.75 ± 36.57*  85.72 ± 53.07*  44.27 ± 18.28  58.74 ± 29.43 | The shoulder strength significantly increased eccentric external work without significant effects on concentric internal total work, concentric internal mean force or eccentric external mean peak force. |

*Continued*

*Continued*

| **Study** | **Outcome measures** | **Results** | | | | **Conclusion** |
| --- | --- | --- | --- | --- | --- | --- |
|  |  | **Intervention** | | **Control** | |  |
|  |  | **Pre** | **Post** | **Pre** | **Post** |  |
| ***Sports Specific*** | | | | | | |
| *Romero-Franco*  *(2012)* | **Eyes Open Results**  **∆ pre/post**  *XEO [mm]  DEO [mm]  SpEO [mm.s^-1^]  YEO [mm]  SEO [cm^2^]  RombergS [index]  RombergSp [index]   RombergD [index]*  **CG control [%]** | -0.78 ± 4.31  90.04 ± 19.71 1.79 ± 0.39 -1.12 ± 6.79 0.62 ± 0.35 1.05 ± 0.59 0.89 ± 0.09 1.23 ± 0.30 53.63 ± 14.40 | | 2.30 ± 2.75* 105.82 ± 23.12 2.14 ± 0.52 2.88 ± 4.76 0.90 ± 0.62 0.74 ± 0.46 0.93 ± 0.09 1.26 ± 0.22 48.18 ± 13.38 | | Slightly improvement of core stability and dynamic parameters such as gravity center control. |
| *Saraswat*  *(2015)* | **T-Test [s]** | 12.4 ± 0.7 | 11.6 ± 0.7”* | 12.1 ± 0.5 | 12.1 ± 0.4 | 4 weeks dynamic balance training significantly improved agility. |

‘’ : Statistically significant difference within groups (pre- post-test).

* : Statistically significant difference between intervention and control group (pre- post-test).

**5JT,** 5 jump test; **BAL**, balance training group; **BESS**, Balance error scoring system; **CG** **control**, general gravity center control; **CMJ**, counter movement jump; **COP**, center of pressure; **DEO**, distance covered by the center of pressure with eyes open; **DJ**, drop jump; **ECC**, Eccentric strengthening exercises; **ER**, external rotation; **IR**, internal rotation; **MAS**, maximal aerobic speed; **MSFT,** multi-stage fitness test; **PLYO**, plyometric training group; **RAST**, running-based anaerobic sprint test; **RJ**; rebound jump,; **RombergD**, Romberg index about distance; **RombergS**, Romberg index about surface; **RombergSp**, Romberg index about speed; **SE**, session(s); **SEBT**, Star Excursion Balance Test; **SEO**, surface covered by the center of pressure with eyes open; **SLH**, single leg hop**; SLTH,** single leg triple crossover hop; **SLS**, single leg squat; **SpEO**, speed of center of pressure movement with eyes open; **THDT**, Triple hop distance; **TTS,** time to stabilization; **UNS**, unstable surface exercises; **VDJ**, vertical drop jump; **VO_2max_**, maximal oxygen uptake; **XEO**, mean position center of pressure in the medial-lateral plane with eyes open; **YEO**, mean position center of pressure in the anterior-posterior plane with eyes open; **YYIRTL1**, Yo-Yo intermittent recovery test level 1;

Values presented as mean ± standard deviation or **∆%** or if not otherwise stated.
